# From Infrared Spectra to Macroscopic Mechanical Properties of sH Gas Hydrates through Atomistic Calculations

**DOI:** 10.3390/molecules25235568

**Published:** 2020-11-27

**Authors:** Shaden M. Daghash, Phillip Servio, Alejandro D. Rey

**Affiliations:** Department of Chemical Engineering, McGill University, Montréal, QC H3A 0C5, Canada; shaden.daghash@mail.mcgill.ca (S.M.D.); phillip.servio@mcgill.ca (P.S.)

**Keywords:** structure-H (sH) gas hydrate, IR spectra, vibrational frequencies, DFT, interatomic distances, bond force constant

## Abstract

The vibrational characteristics of gas hydrates are key identifying molecular features of their structure and chemical composition. Density functional theory (DFT)-based IR spectra are one of the efficient tools that can be used to distinguish the vibrational signatures of gas hydrates. In this work, ab initio DFT-based IR technique is applied to analyze the vibrational and mechanical features of structure-H (sH) gas hydrate. IR spectra of different sH hydrates are obtained at 0 K at equilibrium and under applied pressure. Information about the main vibrational modes of sH hydrates and the factors that affect them such as guest type and pressure are revealed. The obtained IR spectra of sH gas hydrates agree with experimental/computational literature values. Hydrogen bond’s vibrational frequencies are used to determine the hydrate’s Young’s modulus which confirms the role of these bonds in defining sH hydrate’s elasticity. Vibrational frequencies depend on pressure and hydrate’s O···O interatomic distance. OH vibrational frequency shifts are related to the OH covalent bond length and present an indication of sH hydrate’s hydrogen bond strength. This work presents a new route to determine mechanical properties for sH hydrate based on IR spectra and contributes to the relatively small database of gas hydrates’ physical and vibrational properties.

## 1. Introduction

Gas hydrates are one of the available energy resources with a high content of natural gas that drives their exploitation and utilization in different applications. The high capacity is one of the features of gas hydrates that qualifies them for potential use in gas sequestration applications [1,2,3]. Structure-H (sH) gas hydrate is one of the three canonical types of gas hydrates (sI, sII, sH) that is identified by its hexagonal crystal symmetry and anisotropy, which impose a strong directional dependency on its properties [4]. A unit cell of this hydrate consists of one large icosahedron (5^12^6^8^) cage, two medium irregular dodecahedron (4^3^5^6^6^3^) cages, and three small pentagonal dodecahedron (5^12^) cages formed by 34 hydrogen bonded water molecules [5].

Gas hydrates have different applications related to the science and engineering of energy and the environment, the latter due to the fact that they can also present environmental and geological risks [6]. Characterizing and understanding their material properties are crucial for process design, optimal utilization, and proper handling of these materials. Different studies [4,7,8,9,10,11,12,13] on the physical properties of sH gas hydrates are available in the literature with some of them having been undertaken using first-principles computations. However, the complete determination and full atomistic-level understanding of the key properties and characteristics of sH gas hydrates are at present necessary.

There are several techniques that are widely used to detect, identify, and analyze the structure of materials. One of the important chemical analysis techniques is infrared (IR) spectroscopy, which can be used to reveal fundamental knowledge about the structure being analyzed by providing information on the vibrational characteristics of the structure such as frequency peaks, intensities, and band shape and width [14]. Intermolecular vibrations in gas hydrates reflect several of their properties such as thermal conductivity and heat transport properties [15].

IR spectroscopic studies of water are of great interest to research involving gas hydrates [16]; however, it presents a challenge to the field of computational spectroscopy [17]. IR spectroscopic studies of gas hydrates involve studies of molecular interactions, kinetic and structural analysis [16]. Analyzing the molecular vibrations of gas hydrate structures provides crucial information on the existence of gas hydrates, their composition, and the type of interactions guests have with host water molecules [13]. One of the challenges associated with the vibrational studies of gas hydrates is the shift in guest vibrational frequencies compared to the isolated molecule [18]. Another challenge is the presence of IR inactive vibrations that cannot be detected in an IR spectrum. IR spectroscopy is one of the spectroscopic techniques with growing significance in the field of gas hydrates formation and structural analysis [16]. However, spectral computations of ice and gas hydrates are more complicated than that of gas phase molecules [19]. Experimental IR spectroscopy for structures containing water molecules such as gas hydrates is challenged by the high IR absorptivity of water molecules [16]. In addition, the low frequency region of the IR spectrum of water is not easily available through experiments as compared to the high frequency region [17]. This greatly motivates the use of computation-based IR spectroscopic techniques such as density functional theory (DFT) to understand and characterize the vibrational characteristics of gas hydrates.

Hiratsuka et al. [13] used simulations of molecular dynamics to study the molecular vibrations of methane encapsulated in sI gas hydrate and showed that vibrations differ by cage type and are related to the C-H bond length. Vlasic et al. [20] investigated the vibrational properties of sII gas hydrates using ab initio DFT. Experimental IR studies of gas hydrates were undertaken by Kumar et al. [21] to identify the vibrations of carbon dioxide in different hydrate cages to estimate cage occupancies. Rauh et al. [22] investigated the formation of methane hydrates using experimental infrared spectroscopy and highlighted key vibrational modes of the structure. The vibrational characteristics of THF sII gas hydrates were discussed by Vlasic et al. [23] using ab initio DFT computations. THF hydrates were compared to hydrocarbon gas hydrates in terms of elastic and vibrational properties.

This work uses ab initio DFT atomistic level simulations of sH gas hydrates of 2,2-dimethylbutane (neohexane, NH) and different help gas guests (methane, xenon, and carbon dioxide). Understanding the properties of gas hydrates containing methane is an active area in research that is related to the deployment of gas hydrates in energy-related applications. Gas hydrates of carbon dioxide are of great interest from an environmental point of view [3,24]. Understanding the properties of such hydrates is crucial for their potential use in gas storage solutions.

This work focuses on the computations of the infrared spectrum of different sH gas hydrate structures to analyze and interpret their vibrational characteristics with a focus on host water and guest vibrations and associated functional groups. The aim is to characterize and understand key vibrational information of those systems at equilibrium (0 K, 0 GPa) and under applied hydrostatic pressure. The two main objectives of this work are: (i) the utilization of DFT-based IR technique to compute a wide range set of vibrational microscopic properties of the most important and representative sH gas hydrates, and (ii) to use the computed IR-spectra to compute mechanical properties of sH hydrate structures under various pressures. The atomistic-based relations between IR frequencies and bond strength allow for the computation of sH hydrate’s Young’s modulus, which can then be compared to the one computed using direct DFT energy-strain simulations [12]. This compensates for the experimental drawbacks of IR spectroscopy and those of material’s mechanical testing and establishes a novel pathway to transform molecular information into macroscopic mechanical properties. In the future, it is expected that generating a comprehensive data base of sH gas hydrate IR spectra, as computed here, can eventually be used to identify the actual presence of these materials, the approximate ambient pressure conditions where they are found, and the elastic property estimates, by systematic comparison of field-taken IR data and the computational database.

Temperature effects, other chemical compositions, larger scale systems, and other computational platforms not considered here are beyond the scope of this paper and left for future work. The predictions were subjected to rigorous validation and verification protocols. Comparisons with available experimental and computational data were performed.

The organization of this paper is as follows. Section 2 contains the key outcomes of this work including IR vibrational frequencies of guest and host molecules, the hydrogen bond force constant, and Young’s modulus of systems at equilibrium and under applied pressure. The effect of guest type and pressure on the vibrational characteristics of sH gas hydrates and the role of hydrogen bond strength in defining the hydrate’s Young’s modulus are discussed. Interatomic distances and frequency shift relations are also outlined. The implemented DFT-based IR technique and simulations in addition to the analysis tools are outlined in Section 3. Conclusions and important contributions of this work are highlighted in Section 4.

## 2. Results and Discussion

### 2.1. Structure-H Hydrate of Neohexane

In this work, different sH hydrate structures are investigated for their vibrational characteristics. In addition to the empty metastable structure, four filled systems of 2,2-dimethylbutane (neohexane, NH) with different help gases are studied. Those systems are CH_4_-NH, Xe-NH, CO_2_-NH, and CO_2_-CH_4_-NH. In our previous work [11,12], the properties of several sH hydrate systems were examined. Here, new systems containing CO_2_ as a small guest are also investigated. The mechanical properties of empty, CH_4_-NH, and Xe-NH sH hydrates from first principles are available in the work of Daghash et al. [11,12].

Structure optimization is an important step in DFT computations. After geometry relaxation is completed, further optimization can be carried out by varying the different simulation parameters to obtain a structure that is a true energy minimum. For IR spectra computations, this optimization is essential as computed IR spectra will verify if the structure is a true energy minimum by providing a spectrum that is free from imaginary vibrational frequencies. In this work, further optimization to sH hydrate structures is achieved and structures are verified to be true minima. The structural characteristics of investigated systems in this work are presented in Table 1. Even though sH gas hydrate is of hexagonal crystal symmetry, small deviations from the hexagonal crystal characteristics (a = b ≠ c, 90°, 90°, 120°) are expected during structure optimization and relaxation. The structural features of the empty, CH_4_-NH, and Xe-NH sH hydrates are slightly different from those previously reported (Ref. [11]) due to the additional optimization steps taken for those systems.

The geometrical characteristics of the CO_2_-CH_4_-NH sH hydrate agree with the experimental values reported by Uchida et al. [25] at 113 K. Using CO_2_ as a guest molecule in sH hydrate of neohexane increases the unit cell’s a-lattice constant and reduces its c-lattice constant compared to other filled systems. The interatomic distances of the hydrates’ structures agree with the findings of our previous work [12] on sH gas hydrates. The O···O distance and hydrogen bond length are higher for filled sH hydrates due to the stretching effect that guest molecules have on the hydrate structure. On the other hand, the OH covalent bond is slightly shorter for filled systems. When the hydrogen bond stretches, the OH covalent bond shortens, a generic compensation mechanism that was previously demonstrated for gas hydrates [12,26,27].

### 2.2. IR Spectra of Different sH Hydrate Structures at Equilibrium

**Figure 1:**
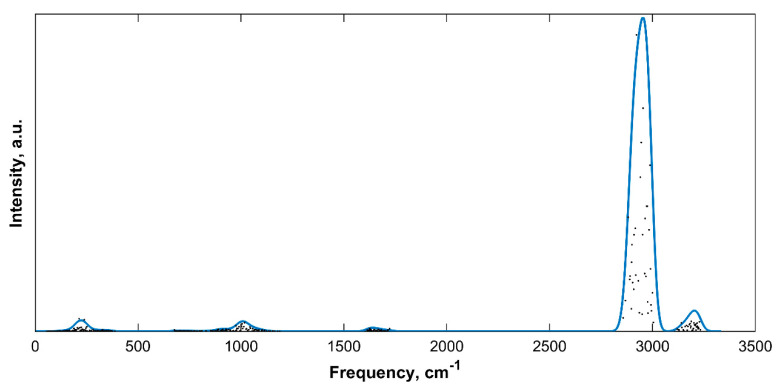
IR spectra of empty sH gas hydrate at equilibrium (0 K, 0 GPa). Gaussian convoluted spectra (line) and raw IR spectra (dots).

The IR spectra of the five sH hydrates investigated in this work were computed at their ground state at 0 K and 0 GPa using ab initio DFT. Figure 1 presents the IR spectrum of empty sH gas hydrate at equilibrium. The main vibrational bands of the hydrogen bonded structure are the hydrogen bond stretching, the H_2_O libration, the H_2_O bending, the OH symmetric stretch, and the OH asymmetric stretch modes.

**Figure 2:**
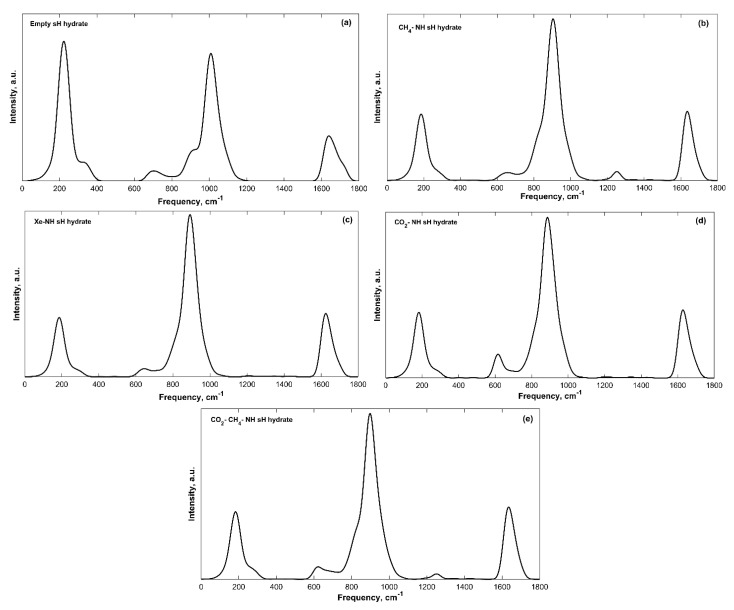
Low frequency convoluted IR spectra bands of sH gas hydrates at 0 K and 0 GPa. (**a**) Empty sH hydrate; (**b**) CH_4_-NH sH hydrate; (**c**) Xe-NH sH hydrate; (**d**) CO_2_-NH sH hydrate; (**e**) CO_2_-CH_4_-NH sH hydrate.

**Figure 3:**
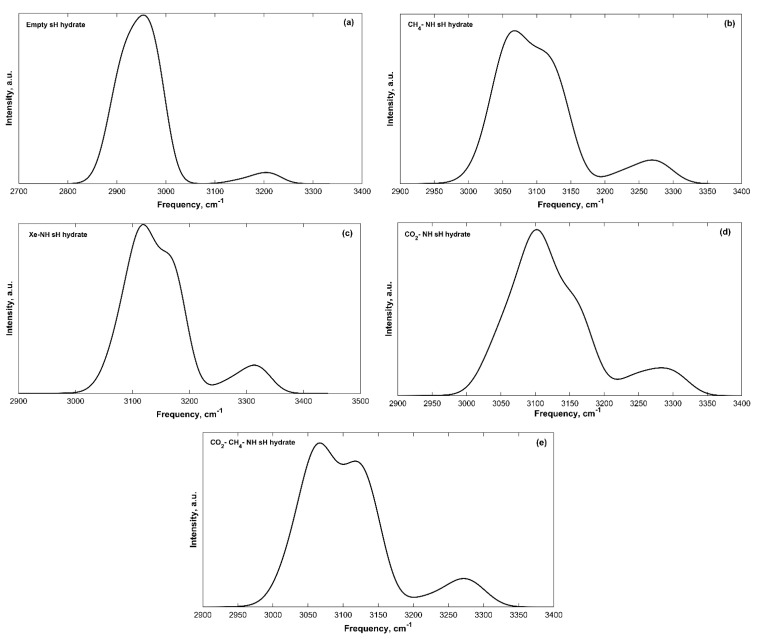
High frequency convoluted IR spectra bands of sH gas hydrates at 0 K and 0 GPa. (**a**) Empty sH hydrate; (**b**) CH_4_-NH sH hydrate; (**c**) Xe-NH sH hydrate; (**d**) CO_2_-NH sH hydrate; (**e**) CO_2_-CH_4_-NH sH hydrate.

The IR spectra of filled sH gas hydrates were also obtained. Band peak assignment was performed based on the knowledge of molecular vibrations of individual molecules and of hydrate structures from literature. The IR spectra of all sH hydrates presented in this work are divided into a low frequency band (**ω** < 2000 cm^−1^) region and a high frequency band (4000 > **ω** > 2000 cm^−^^1^) region. Guest vibrations are shown separately for filled systems as well. The low frequency bands of the convoluted IR spectra of sH gas hydrates are presented in Figure 2 and the high frequency bands are presented in Figure 3.

The hydrogen-bonded host water molecules of sH gas hydrates essentially have five vibrational modes, three related to the vibrations of the water molecule itself and two caused by the hydrogen bonding effect. Hydrogen bond stretching generates the first band with frequency peaks of 182–223 cm^−^^1^ for sH gas hydrates investigated here. There are two libration motion bands for sH gas hydrates, a more intense band with peaks of 886–1009 cm^−^^1^ and a less intense one with peaks of 615–703 cm^−^^1^. H_2_O bending creates the third vibrational band with peaks of 1626–1640 cm^−^^1^. The high frequency bands are those of the OH symmetric stretch with band peaks of 2954–3119 cm^−1^ and OH asymmetric stretch with band peaks of 3204–3314 cm^−^^1^. The equilibrium state (0 GPa) IR peak frequencies are presented in Table 2 for empty and filled sH gas hydrates. The hydrogen bond stretching, the H_2_O libration and bending band peaks are the highest for the empty system compared to the filled systems. On the other hand, sH hydrate filling causes an increase in the peak frequencies of the OH symmetric and asymmetric stretches, compared to empty sH. These observations agree with the findings of Vlasic et al. [20] for IR vibrational frequencies of sII gas hydrates. A good agreement is observed between the frequencies of empty and CH_4_-NH sH hydrates (this work) and empty and CH_4_-C_3_H_8_ sII hydrates [20].

The intensity of vibrational peaks is a distinguishing feature of IR spectra. The availability of different functional groups can be determined using peak intensity [14]. For sH gas hydrates, the OH symmetric stretching band has the highest intensity among all bands, and it is the highest for the empty structure compared to filled sH hydrates. This is expected as the intensity of this band depends on the number of OH bonds in the unit cell. Similarly, the hydrogen bond stretching band intensity is higher for empty sH hydrate. On the contrary, the intensities of the OH asymmetric stretching, the H_2_O libration 2, and H_2_O bending bands are lower in empty sH compared to filled sH hydrates.

The band width is another characteristic of the IR spectra of a material. Depending on the material’s composition and the presence of different functional groups, bands experience varying widths [14]. The hydrogen bond interaction between water molecules in sH hydrates can vary based on the number of bonds and their strength in the studied structure. This kind of variation causes changes to the chemical environment of the hydrate, which leads to a variation in band widths specially when comparing empty to filled sH hydrates. The band widths in sH hydrates investigated in this work were found to slightly differ from one structure to another.

The OH symmetric, asymmetric stretches, and H_2_O bending IR frequencies of water vapor are around 3656, 3755, and 1594 cm^−^^1^, respectively [28,29]. The hydrogen bond stretch and libration modes of water at 0 °C from FTIR are 183.4, 395.5, and 686.3 cm^−^^1^, respectively [30]. Libration bands of ice (Ih) at 21 K were found to be 71 and 91 meV (≈572.7 and 734 cm^−1^) [31]. Comparing these values to band peaks of empty sH gas hydrates shows the blueshift in hydrogen bond stretching, H_2_O libration, and H_2_O bending bands from that of vapor and liquid water. On the other hand, a redshift—from that of vapor water—of the OH symmetric and asymmetric stretch bands is observed. This shift in spectral band peaks of water molecules is due to increased hydrogen bonding in hydrates. This shift has been previously confirmed for ice [31,32], gas hydrates [20], and other materials [33,34]. The OH stretching frequencies of water can experience significant redshifts as water condenses in structure. This redshift is known in condensed water molecule systems and can be as much as 1000 cm^−1^ depending on the environment [35]. The hydrogen bonding in condensed forms of water causes softening of OH bonds stretching [32]. Using water vapor as a basis and computing the change in frequency for sH gas hydrates reveals interesting findings. As hydrate volume increases, the concentration of hydrogen bonds decreases [11], which causes the frequency shift (from H_2_O vapor) to be smaller. This is reflected in the OH stretching frequencies of filled sH hydrates that are closer to those of the vapor H_2_O than the empty sH hydrate is. The redshift (from water vapor) of the OH symmetric stretching band is higher than that of the OH asymmetric stretching band for all systems at equilibrium and under pressure, which agrees with the findings of Choi and Cho [36].

The Raman peak of OH symmetric stretch of CH_4_ sII gas hydrate was found to be 3450 cm^−1^, while that of CH_4_ sI gas hydrate and ice (Ih) are around 3100 cm^−1^. A peak at around 210 cm^−1^ was assigned for the hydrogen bond stretching band of sI hydrate and ice (Ih) [37]. A DFT study [38] showed that the low frequency vibrational modes of ice (Ih) are in good agreement with those obtained for sH gas hydrates, with some differences related to structures and simulation parameters. Vlasic et al. [20] compiled some literature findings on vibrational frequencies of different structures of gas hydrates and ice.

#### Effect of Filling and Guest Type on the IR Spectra of sH Hydrate

Using empty sH gas hydrate as a basis makes it possible to understand the effect of guest molecules on sH hydrate by observing variations in its IR spectra. Upon cage filling with guest molecules, the hydrogen bond stretching, and H_2_O libration band peaks are redshifted to lower frequency values compared to the reference empty sH gas hydrate. The redshift in hydrogen bond stretching frequencies is higher compared to that of H_2_O libration and bending bands. The H_2_O bending band peak frequency experiences small variations (less than 1%) with sH hydrate filling, which implies that this band is not affected by the presence of guest molecules. On the other hand, OH stretching bands of filled sH hydrates are blueshifted to higher frequency values compared to empty sH. The increase in frequency values is higher for the OH symmetric stretch compared to the OH asymmetric stretch. The degree of a band’s frequency shift depends on the type of guest. For example, the presence of methane in sH gas hydrate cages lowers the degree of blueshift of OH stretching frequencies. However, encapsulation of xenon causes the highest blueshift in OH stretching bands’ frequencies. This emphasizes the importance of addressing guest-host molecular interactions in the interpretation of sH hydrate IR spectra.

**Figure 4:**
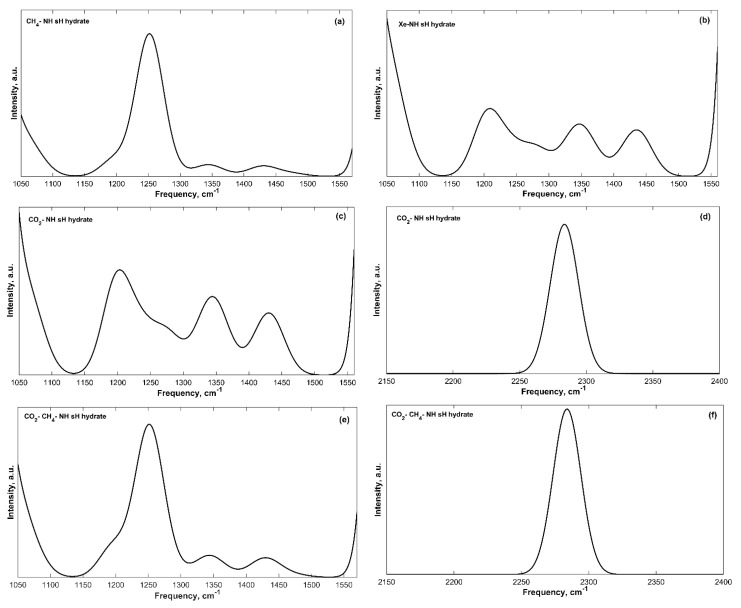
Guest vibration bands from convoluted IR spectra of filled sH gas hydrates. (**a**) CH_4_-NH sH hydrate; (**b**) Xe-NH sH hydrate; (**c**) CO_2_-NH sH hydrate; (**d**) CO_2_-NH sH hydrate (CO_2_ asymmetric stretch band); (**e**) CO_2_-CH_4_-NH sH hydrate; (**f**) CO_2_-CH_4_-NH sH hydrate (CO_2_ asymmetric stretch band).

The guest molecules’ vibrations introduce additional bands to the IR spectra of sH gas hydrates. These vibrations are less intense compared to those of the hydrogen bonded water molecules. Guest molecules have different IR active vibrations; however, only some of them were observable in the IR spectra of sH hydrates either because they are too weak or because they are blended with host water vibrations such as CH_3_ symmetric or asymmetric stretches and C-H bond stretch. The peak assignment of the guest vibrations is presented in Table 3 and some guest bands are shown in Figure 4. The assignment was based on available knowledge of individual molecular vibrations in literature for 2,2-dimethylbutane (neohexane) [39,40,41], methane [13,28,42], and carbon dioxide [21,28,43] molecules.

Neohexane experiences four different types of IR active vibrations that were observed in our IR spectra of neohexane sH hydrates. The first one has a peak in the range of 474 and 483 cm^−1^ and is caused by the carbon-carbon-carbon bend. The C-C-C bending vibrations of neohexane inside sH hydrate are very close to that of the isolated molecule [39,40]. For the CH_4_-NH, Xe-NH, CO_2_-NH, and CO_2_-CH_4_-NH sH hydrates, additional peaks at 408, 406, 404, and 408 cm^−1^, respectively, are observed for this band. The CH_3_ groups of neohexane have rocking vibrations with peaks in the frequency range of 1196 to 1209 cm^−1^. The same groups have symmetric bending vibrations (1344–1347 cm^−1^) that have lower energy than the asymmetric bending vibrations (1430–1435 cm^−1^).

Methane molecules have different IR active vibrations; however, only some of them were observable in the IR spectra of sH hydrates containing methane. These are the CH_4_ bending vibrations (1251–1252 cm^−1^), which are more intense than the CH_4_ rocking vibrations (1478 cm^−1^). The C-H symmetric stretch of methane is IR inactive. MD vibrational study [13] of the spectra of sH hydrate containing methane shows that the CH_4_ bending peaks are in the range of 1252.7–1257.5 cm^−1^, while the CH_4_ rocking peaks are in the range of 1457.9–1465.6 cm^−1^. Vlasic et al. [20] found the CH_4_ rocking vibration frequencies at 0 K to be equal to 1475 cm^−1^ in ethane-methane and propane methane sII gas hydrates. Our findings agree with the results of these studies. Molecular rotation and translation of methane inside sH hydrate cages were also reported [13]; however, these vibrations are in the same range of the transition band and cannot be observed in our IR spectra.

The IR active vibrations of carbon dioxide are the CO_2_ bending and CO_2_ asymmetric stretch (symmetric stretch is IR inactive). These two vibrational modes were observable in our IR spectra of sH hydrates containing carbon dioxide. However, at equilibrium state (0 GPa), only the CO_2_ asymmetric stretch vibrations were distinguishable in the IR spectra. The obtained peak frequency of this band is 2284 cm^−1^. An FTIR study [43] of sII hydrates of propane and carbon dioxide at −50 °C has shown that this band has a peak at 2346 cm^−1^, which is close to the CO_2_ vibration in the gas phase (2349 cm^−1^) [28]. Another experimental IR study [44] of CO_2_ hydrates of structure I at 13 K has reported CO_2_ asymmetric stretch vibrations at 2347 and 2334 cm^−1^. The reported peak of the CO_2_ asymmetric stretch in this work is smaller by 50 to 62 cm^−1^, which could be due to difference in techniques (theoretical/experimental), temperature, and hydrate structure.

**Figure 5:**
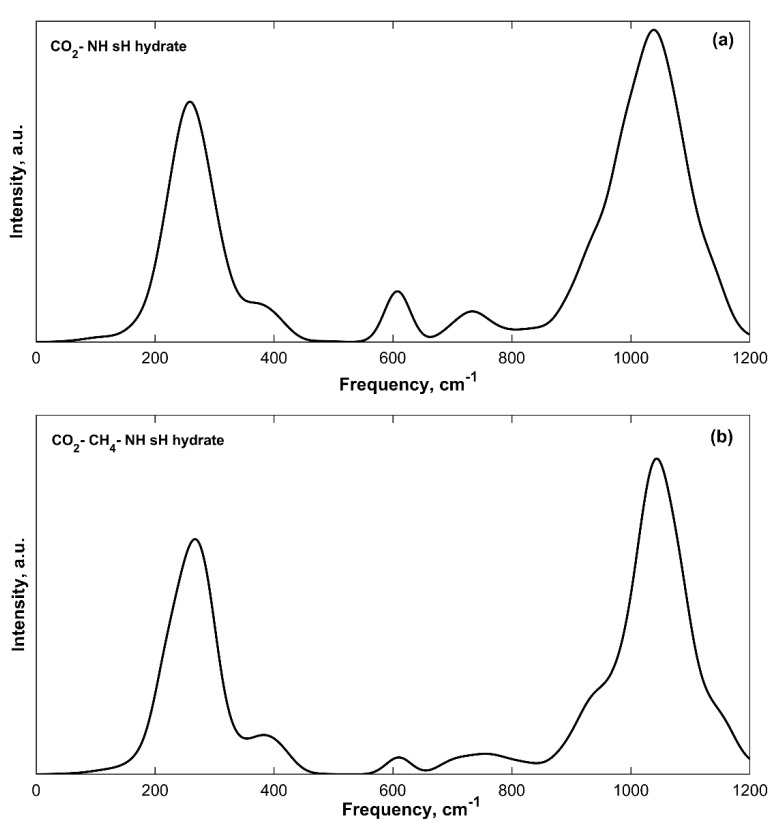
CO_2_ bending band observed at 607 cm^−1^ (**a**) and 610 cm^−1^ (**b**) in sH gas hydrates at 3 GPa.

The CO_2_ bending vibrations of isolated CO_2_ gas [28] have a peak at around 667 cm^−1^. At 0 GPa, this vibrational band is not observable in the IR spectra of sH hydrates as it is blended with the first H_2_O librational band that has peak frequencies of 615–657 cm^−1^ for filled sH hydrates (Table 2). The CO_2_ bending band is clearly observed when sH hydrate is under compression as the H_2_O librational bands get blueshifted to higher frequency values (as will be explained later). Figure 5 presents the low frequency region at 3 GPa in CO_2_-NH (a) and CO_2_-CH_4_-NH (b) sH hydrates. The CO_2_ bending band is observed with peaks at 607 cm^−1^ (CO_2_-NH) and 610 cm^−1^ (CO_2_-CH_4_-NH) when both systems are under compression of 3 GPa. Fleyfel and Devlin [44] reported this band peak from experimental IR (at 13 K) at 655 and 656 cm^−1^ in CO_2_ sI and sII gas hydrate, respectively.

### 2.3. Bond Strength and Hydrate’s Young’s Modulus from IR Spectra

In addition to the structure identity obtained from the IR spectra of sH gas hydrates, the vibrational frequencies of main vibrational modes can be of great use in reflecting sH hydrate bonds’ strength and mechanical properties. By visualizing the hydrogen bond as string connecting a hydrogen atom and an oxygen atom, it is possible to determine the strength of the hydrogen bond using Hooke’s law and a simple harmonic oscillator model (see Equation (1) [14] below). Here, **ω** is the vibrational frequency (cm^−1^), **c** is the speed of light (cm·s^−1^), **M_1_** is the mass of the hydrogen atom (kg), **M_2_** is the mass of the oxygen atom (kg), and **k** is the spring or the bond force constant (N.m^−1^). Equation (1) relates the hydrogen bond force constant to its vibrational frequency. A higher frequency reflects a stronger hydrogen bond. Equation (1) does not account for the anharmonic motion in real vibrations of molecules, so the computation made using this equation is an approximation [14]. The accuracy of this approximation is established posteriorly with direct DFT predictions of elastic moduli and with existing measurements.

Computed hydrogen bond constants can be utilized in estimating Young’s modulus of sH gas hydrates using Equation (2) [45] below. Here, **E** is hydrate’s Young’s modulus, **k** is the hydrogen bond’s force constant, and **R_O_** is the hydrate’s average hydrogen bond length at equilibrium (0 GPa). This equation provides a simple and a direct assessment of the material’s Young’s modulus using the knowledge of its bond force constant and bond length at equilibrium. The hydrogen bond has been previously [11,20,46] shown to be an important factor in determining many gas hydrates’ properties and hence it is used to estimate the Young’s modulus here using infrared frequencies.

(1) $$\omega =\frac{1}{2\pi c}\sqrt{\frac{k\left(M_{1}+M_{2}\right)}{M_{1}M_{2}}}$$

(2) $$E=\frac{k}{R_{o}}$$

The computed hydrogen bond force constant and sH hydrate Young’s modulus are presented in Table 4. The hydrogen bond of the empty system is the strongest among all systems investigated here. Filling of sH gas hydrates weakens the hydrogen bonds of the structure. Filled sH hydrates have an average (**k**) of 1.897 N.m^−1^, which is around 32% less than that of empty sH hydrate. This proves the effect of guest molecules on sH gas hydrates’ properties, which was previously determined [11,12] and reflects the guests’ role in weakening the hydrogen bonds of the hydrate structure and hence reducing its stiffness. Empty sH hydrate has the highest hydrogen bond force constant as its hydrogen bonds vibrate at the highest hydrogen bond stretching frequency presented in this work.

The strength of the hydrogen bond in filled systems is affected by the guest type and hence guest-host interactions. Both of CH_4_-NH and Xe-NH sH hydrates have equal hydrogen bond’s force constants, which are higher than those of systems containing CO_2_. The presence of CO_2_ in sH hydrate weakens the hydrogen bonds of the hydrate structure. This might be related to the linear shape of the CO_2_ molecule and the type of interactions it has with neighboring water molecules. Computing Young’s modulus using hydrogen bond information shows that empty sH hydrate is stiffer than other systems investigated here, and that Young’s modulus decreases with hydrate filling. The same was observed by Vlasic et al. [20] for sII gas hydrates. The obtained hydrogen bond force constants and Young’s modulus of sH gas hydrates are close to those reported by Vlasic et al. [20] for sII gas hydrates.

Young’s modulus is one of the important mechanical properties that can be obtained using the crystal’s elastic constants and polycrystalline approximations. The energy-strain analysis is one of the established methods in obtaining the material’s elastic constants based on its crystal symmetry. This approach had been previously used to compute the elastic constants and Young’s modulus of several sH gas hydrates including the empty, CH_4_-NH, and Xe-NH sH hydrates [12]. Young’s modulus values from elastic constants of these systems are presented in Table 4 for comparison. Young’s modulus of sH gas hydrate computed from elastic constants is generally higher for filled systems compared to empty sH [12]. However, infrared-based Young’s modulus is the highest for the empty sH hydrate due to its higher hydrogen bond stretching frequency. Nevertheless, the two methods provide Young’s modulus values that are of the same order of magnitude. Results from infrared analysis emphasize the critical role of the hydrate’s hydrogen bonds in determining sH gas hydrate properties. It reflects on the embedded mechanical characteristics in the infrared spectrum, which can be extracted and utilized. The hydrogen bond was previously found to contribute to sH gas hydrate’s bulk modulus [11] and in this work its strength reflects sH hydrate’s stiffness. Using sH gas hydrate’s hydrogen bond characteristics to obtain its Young’s modulus is complementary to the well-established elastic constants method. Where measurements of elastic constants are inaccessible, infrared spectra of gas hydrate can be utilized to reflect on its stiffness, composition, and pressure conditions.

The values of force constant (and hence Young’s modulus) are sensitive to the hydrogen bond stretching frequency value. An increase of 10 cm^−1^ in this frequency for the CO_2_-NH system at equilibrium can increase the Young’s modulus by 1.16 GPa or 11%. In addition, excluding anharmonicity from IR computations has its own impact on obtained vibrational frequencies and hence hydrogen bond force constant.

Values of hydrogen bond’s length (Table 1) and hydrogen bond’s force constant (Table 4) show that a shorter hydrogen bond is not necessarily stronger. The hydrogen bond of the Xe-NH sH hydrate at equilibrium is longer than that of the CO_2_-CH_4_-NH; however, the bond force constant of the hydrogen bond in Xe-NH sH hydrate is higher. The CH_4_-NH sH hydrate has a shorter hydrogen bond compared to the Xe-NH sH hydrate; however, both systems have the same hydrogen bond force constant as their hydrogen bonds stretch at the same frequencies. There are two factors that determine the hydrogen bond’s strength, which are its length and its associated angle. The longer the hydrogen bond is and the more its associated angle deviates from 180°, the weaker it is. Encapsulation of guest molecules inside sH hydrate cages introduces a certain level of lattice distortion that weakens their hydrogen bonds. The same observation was made by Vlasic et al. [20] for sII gas hydrates.

The molecular weight of the guest molecules encapsulated inside sH gas hydrate impacts its hydrogen bond force constant and hence its IR-based Young’s modulus. Results of the CH_4_-NH, CO_2_-NH, and CO_2_-CH_4_-NH sH hydrates show a decrease in force constants and Young’s modulus with increased average molecular weight of the guests. The Xe-NH sH hydrate is excluded from this observation as despite the large molecular weight of xenon, the hydrogen bond force constant and Young’s modulus of Xe-NH sH hydrate are not the lowest compared to other systems. Xenon is a single atomic gas that does not contain bonds and hence does not absorb IR radiation [14]. Due to this, the Xe-NH sH hydrate can be excluded from this remark. However, to better understand the effect of guest molecular weight, more than three systems need to be considered.

### 2.4. Effect of Hydrostatic Pressure on IR Spectra

The effects of applied hydrostatic pressure (tensile and compression) on the IR spectra of sH gas hydrates are discussed in this section. The IR spectra under pressure reveal different information about the material, which are related to its microscopic vibrational and macroscopic mechanical characteristics.

#### 2.4.1. Vibrational Frequencies and Intensities under Pressure

**Figure 6:**
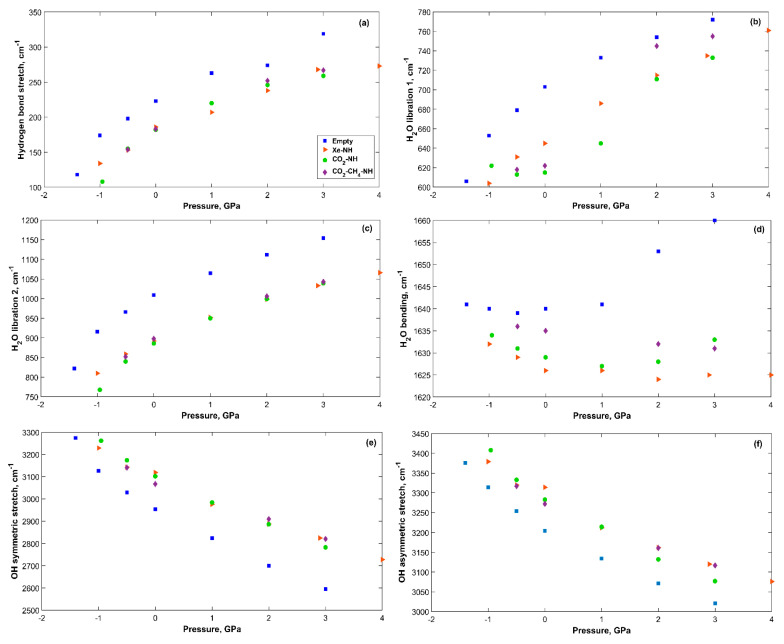
Vibrational modes of H_2_O in sH gas hydrates (see Supplementary Materials) vs. applied pressure. (**a**) Hydrogen bond stretch; (**b**) H_2_O libration 1; (**c**) H_2_O libration 2; (**d**) H_2_O bending; (**e**) OH symmetric stretch; (**f**) OH asymmetric stretch.

The vibrational modes of sH gas hydrates are affected by applied tensile and compression stress. Figure 6 shows the main vibrational modes of the hydrogen bonded water molecules in sH hydrate structures under pressure. The hydrogen bond stretching frequency and the H_2_O librational vibrations increase with increased pressure. On the other hand, H_2_O bending and OH stretching frequencies decrease with applied pressure. To understand the behavior of vibrational frequencies under pressure, it is useful to link them to the corresponding bonds and their response to pressure. Under compression, the hydrogen bond decreases in length, while the OH covalent bond length increases in gas hydrates as was previously proven for sI [27], sII [26], and sH [12] gas hydrates. The shorter the hydrogen bond, the stronger it is (for the same system). A stronger bond leads to a higher vibrational frequency and that explains the increase in the hydrogen bond stretching frequency with pressure. The opposite is true for the OH covalent bond that becomes weaker resulting in lower OH stretching frequencies with increased pressure.

H_2_O librational modes of vibration are related to the hydrogen bonds of the system [47]. This explains the increase in H_2_O librational band frequencies with increased pressure. H_2_O bending vibrations are less affected by applied pressure than other vibrational bands of the hydrogen bonded H_2_O molecules. For filled systems, this band’s frequencies tend to decrease with increased pressure. The reason could be related to the restricted motion of H_2_O molecules under compression, which reduces vibrational frequencies from H-O-H bend. For empty sH hydrate, this band’s frequency decreases with increased pressure in the tensile region, but it increases in the compression region. This is a unique behavior compared to that of filled systems. The behavior of the H_2_O vibrational modes under pressure in sH hydrates agrees with that reported for sII gas hydrate [20].

Applied pressure also affects the band shapes and widths. Close frequency range bands become more separated under compression and become more blended near the maximum tensile strength of sH hydrate. This affects the recognition of guest vibrational bands that are in the same region of H_2_O vibrational bands at equilibrium. The tensile strength of several sH gas hydrates is provided in reference [12]. For example, the CO_2_ bending band is only obvious when systems containing CO_2_ are under compression, as this band has a peak frequency that falls in the H_2_O libration 1 band. Guest vibrations experience less variations with pressure compared to those of the H_2_O molecules that form the hydrate structure. The shift in H_2_O molecules’ vibrations bands allows for some guest bands to be seen. For the Xe-NH and CO_2_-NH sH hydrates, some vibrational peaks related to the neohexane molecule can only be observed when systems are near their tensile strength. For the Xe-NH sH hydrate at −1 GPa, vibrations in the range of 2950 to 3074 cm^−1^ appear with a peak at around 3062 cm^−1^. For sH hydrate of CO_2_-NH at −0.955 GPa similar vibrations are observed in the range of 2941 and 3060 cm^−1^ with a peak at around 3053 cm^−1^. Mirkin and Krimm [40] had assigned a peak at 2965 cm^−1^ for CH_3_ asymmetric stretch in neohexane. Synder and Schachtschneider [39] had assigned a peak at 2964 cm^−1^ for CH asymmetric stretch in CH_3_ groups of neohexane. Our observed peak is close to these values and can be assigned to CH_3_ asymmetric stretch of neohexane molecule encapsulated inside the large cage of sH gas hydrates. Differences in values could be related to the simulation parameters, specifically those defining the guest-host molecular interactions. The interactions that neohexane has with neighboring H_2_O molecules in the hydrate structure affects its vibrational frequencies, which was observed for its other vibrations reported at equilibrium (Table 3).

Even though guest molecules’ vibrations are less affected by applied pressure compared to those of H_2_O molecules, there is a small change in their frequencies as pressure changes. The C-C-C bending in neohexane has two peaks in the pressure range (−1 to 1 GPa). Both peak frequencies increase with pressure and merge into one band at pressure >1 GPa. For Xe-NH sH hydrate at pressure >3 GPa, this band falls into the hydrogen stretching band.

The CH_2_/CH_3_ rocking, CH_3_ symmetric bend, and CH_3_ asymmetric bend frequencies of neohexane increase with pressure. The CH_4_ bending and rocking frequencies also increase with increased pressure. Similarly, the CO_2_ asymmetric stretch increases with applied pressure; however, the CO_2_ bending vibration frequency decreases with applied pressure. The CH_2_/CH_3_ rocking band becomes merged with other bands and cannot be identified in the IR spectra of sH hydrates at pressures >1 GPa for Xe-NH and CO_2_-NH sH hydrates, and at pressures >2 GPa for CO_2_-CH_4_-NH sH hydrate. This band is merged with the CH_4_ bending band in the systems containing methane, which makes it less observable in sH hydrates containing methane. The CH_3_ asymmetric bend was found to be more affected by applied pressure compared to the CH_3_ symmetric bend. Among all guest vibrations, the CO_2_ asymmetric stretch band is the most affected by applied pressure. The dependence of guest molecules’ vibrations on pressure is shown in Table 5.

Vibrational mode intensities are affected by applied pressure as well. The OH symmetric stretch and hydrogen bond stretching intensities increase with applied pressure for empty sH hydrate. However, the change in intensity of those bands in filled systems and of the H_2_O bending and librational bands in all systems varies depending on the sH hydrate under study.

#### 2.4.2. Change in Bond Strength and Young’s Modulus with Pressure

Applying pressure on sH gas hydrate affects this hydrogen bonded network in different aspects. The principal effect is that observed on interatomic distances, which are the hydrogen bond length, the covalent OH bond length, and the oxygen-oxygen distance. These were previously studied under pressure for sH [12], sII [26], and sI [27] gas hydrates from first principles. In this work, the effect of pressure on the investigated sH hydrates is demonstrated through the change in hydrogen bond strength and Young’s modulus computed from IR vibrations. Table 6 lists the pressure dependent hydrogen bond force constant and Young’s modulus for all investigated systems. For each sH hydrate structure, an increase in pressure means a decrease in the hydrogen bond length (see Ref. [12]), which means a stronger bond that vibrates at a higher frequency (for the same system). Stronger hydrogen bonds lead to higher Young’s modulus and more resistance to applied stress for sH gas hydrates.

**Figure 7:**
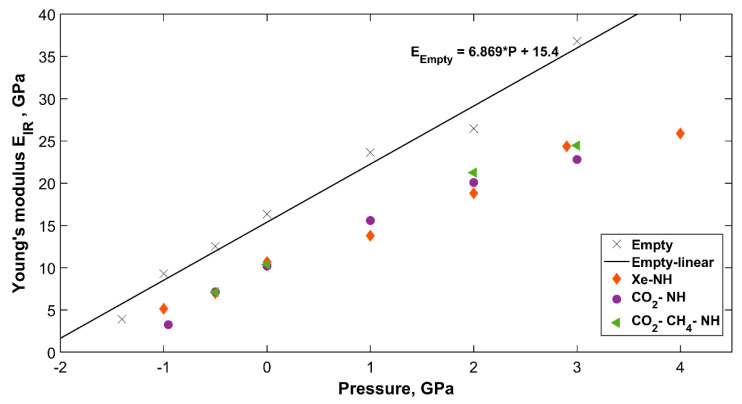
IR computed sH hydrate’s Young’s modulus as a function of pressure.

The pressure dependent Young’s modulus for all sH hydrates is presented in Figure 7. Empty sH hydrate’s Young’s modulus shows the highest dependency on pressure compared to the filled systems. Vlasic et al. [20] presented a similar relationship for sII gas hydrate’s Young’s modulus, which agrees with the findings here for sH hydrate. The results of sH gas hydrates (present work) and sII [20] gas hydrates reflect the higher dependency of empty hydrate’s Young’s modulus on pressure compared to filled hydrate structures. The CO_2_-CH_4_-NH sH hydrate has the highest dependency of Young’s modulus on pressure among “filled” sH hydrates investigated here. The CO_2_-NH follows, and the Xe-NH sH hydrate has the lowest dependency of its Young’s modulus on applied hydrostatic pressure. This proves the effect of guest molecules type and shape on sH hydrate properties and strength [12].

Future work can explore the complete evaluation of sH gas hydrate’s elastic constants’ variations with applied pressure. This will provide essential physical insights on the pressure dependency of the hydrate’s bulk, shear, and Young’s moduli, Poisson’s ratio, and speeds of sounds. Pressure dependent Young’s modulus from elastic constants can be compared to the IR-based results presented here to better understand the strengths and limitations of using hydrogen bonds’ vibrational characteristics to obtain sH gas hydrate’s Young’s modulus.

#### 2.4.3. Vibrational Frequency vs. Interatomic Distances

The interatomic distances in the sH hydrate structure were found to be related to the molecular vibrational frequencies. In the literature, several efforts have been made to present and analyze these relations for different materials [20,33,34,48,49]. For the sH gas hydrates studied in this work, several relations are found between the interatomic distances in the hydrate structure and its main vibrational modes. A link between frequency shifts and hydrogen bond strength is also established in this work using sH hydrate IR spectra at equilibrium and under tensile and compressive hydrostatic pressure.

**Figure 8:**
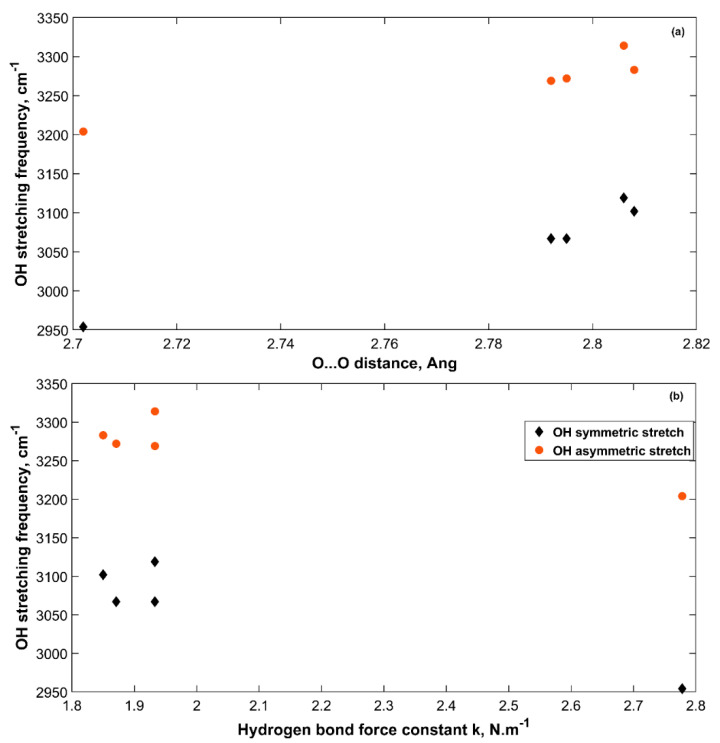
OH stretching frequencies versus O···O distance (**a**) and hydrogen bond force constant (**b**) in sH gas hydrates at zero pressure (0 GPa).

Figure 8 presents the change in OH symmetric and asymmetric stretching frequencies with O···O distance for the five sH hydrates studied in this work at zero pressure. The longer the O···O distance, the higher the symmetric and asymmetric OH stretching frequencies. This is due to the reduced hydrogen bonding effect that gets smaller as the O···O distance gets longer. This causes a reduction in the redshift of these stretching frequencies from those of water in the gas phase. Figure 8 shows how the weaker the hydrogen bond of sH hydrate is (smaller **k**), the higher the OH stretching frequencies are.

**Figure 9:**
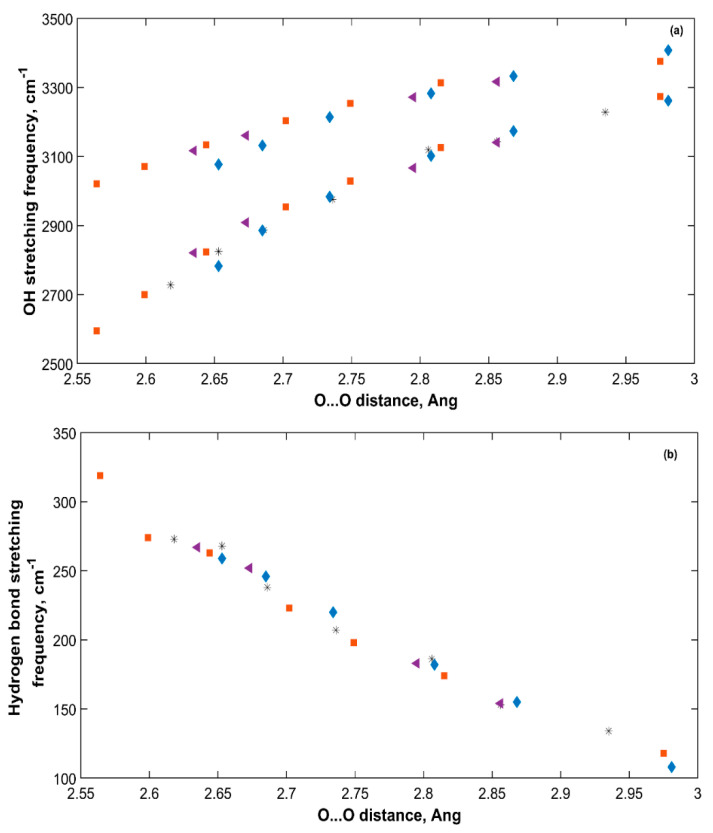
OH (**a**) and hydrogen bond (**b**) stretching frequencies of all sH hydrates versus O···O distance. Empty (■), Xe-NH (*), CO_2_-NH (♦), CO_2_-CH_4_-NH (◄).

The OH and hydrogen bonds stretching frequencies under applied pressure are plotted versus the corresponding O···O distances of all sH hydrates in Figure 9. The OH stretching frequencies of an sH hydrate under pressure increase with increased O···O distance, which agrees with the observation made by Figure 8 at equilibrium. In hydrogen bonded systems, a correlation between OH stretching frequencies and O···O distance has been previously discussed in literature for sII gas hydrates [20], and minerals [50], and similarly between OH stretching frequencies and O···O distance in THF clathrate hydrates [34]. Our results of sH gas hydrates’ vibrations agree with these findings. The decrease in the hydrogen bond stretching frequency with increased O···O distance implies a direct relationship between the hydrogen bond force constant (**k**) and the O···O distance of the hydrate structure.

#### 2.4.4. sH Hydrate Vibrational Frequency Shifts

**Figure 10:**
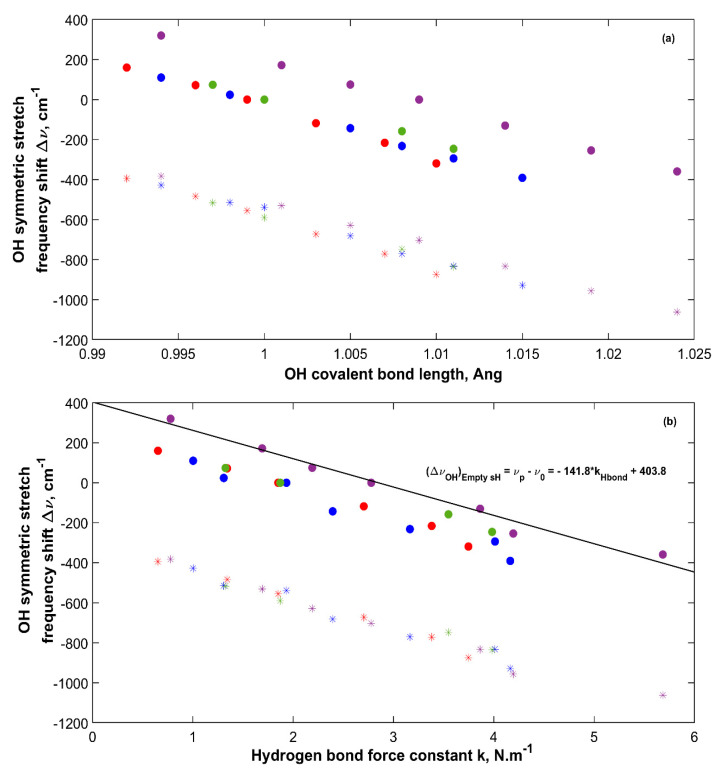
OH symmetric stretching frequency shift Δν of sH hydrates versus OH covalent bond length (**a**) and versus hydrogen bond force constant (**b**). (●) computed shift with system at equilibrium as a reference. (*) computed shift with water vapor as a reference. Color code: empty sH (purple), Xe-NH sH (blue), CO_2_-NH sH (red), CO_2_-CH_4_-NH sH (green).

Vibrational frequencies of hydrogen bonded water molecules in structures such as gas hydrates are affected by different factors such as pressure and structure filling and composition. These factors affect the hydrogen bond and the OH covalent bond, which cause a shift in their vibrational frequencies. It is of interest to observe how the OH vibrational frequencies of water molecules in sH hydrate shift with respect to a reference state. In this work, two references are considered, which are the sH hydrate itself at equilibrium (0 GPa) and water molecules in the vapor phase. Figure 10 presents the shift in OH bond symmetric stretching frequency versus the OH covalent bond length (a) and the hydrogen bond force constant (b). Taking sH hydrate at equilibrium as a reference and computing Δν shows that as the OH covalent bond length decreases (under tensile), the OH symmetric stretch blueshifts to higher vibrational frequencies. The opposite is true for the system under compression at which the OH covalent bond length increases and hence the bond itself weakens leading to a redshift in its vibrational frequency compared to the equilibrium state (0 GPa).

Figure 10 also presents the OH frequency shift from water vapor (Ref. [28,29]) for sH hydrates. All values present a redshift as the OH vibrational frequency is higher for water in the vapor phase and gets reduced by the hydrogen bond effect as water transforms into a condensed phase (i.e., gas hydrates). The degree of this redshift is higher for longer OH covalent bonds (hydrate under compression) due to the abovementioned reasons. The relationship between OH frequency shift and OH bond length in Figure 10 agrees with the findings of Gu et al. [33]. Figure 10b presents a direct relationship between OH frequency shift and hydrogen bond force constant in sH gas hydrates. This agrees with the results of Li et al. [47], who relates the hydrogen bond strength to the redshift of the X-H stretching frequency. Figure 10 supports the use of OH stretching frequency in sH hydrate as a measure of its hydrogen bond strength. The OH asymmetric stretching frequency shift has a similar behavior as the one presented in Figure 10 for OH symmetric stretching frequency.

## 3. Methods

The outcomes of this work are based on the ab initio DFT simulations of five sH hydrate structures. An empty structure and four structures of 2,2-dimethylbutane (neohexane, NH) encapsulated inside the large cage (5^12^6^8^), with xenon, methane, and carbon dioxide encapsulated inside the medium (4^3^5^6^6^3^) and the small (5^12^) cages of sH hydrate were investigated. For the mixed system of CO_2_-CH_4_-NH, methane molecules occupy the small cages, while carbon dioxide molecules occupy the medium ones of sH hydrates based on literature studies [25] on the preferred occupancy of CO_2_ and CH_4_ in sH hydrate cages. Even though CO_2_ fits into the small and medium cages of sH hydrates [25,51,52], it has higher preference to occupy the medium cages—in the presence of methane—due to its non-spherical shape.

A unit cell of 34 hydrogen bonded water molecules of one large, two medium, and three small cages was simulated using the Spanish Initiative for Electronic Simulations with Thousands of Atoms (SIESTA) [53]. The unit cell with 100% single guest occupancy was initially generated using MOLDEN [54]. The initial geometrical coordinates of a zero-dipole moment unit cell provided by Okano and Yasuoka [55] were optimized in our previous work [11,12] and were used in this work as an initial geometrical input. Okano and Yasuoka [55] placed the hydrogen atoms following ice rules to obtain the lowest potential energy unit cell.

DFT computations using Kohn-Sham equations were performed at 0 K to optimize the sH hydrate structures investigated in this work. A supercell size of 2 × 2 × 2 was generated using periodic boundary conditions and the total system’s energy was minimized using structure relaxation. The exchange-correlation (XC) functional that was used is the Generalized Gradient Approximation (GGA) revised Perdew−Burke−Ernzerhof (revPBE) [56], which has been successfully used for DFT computations involving gas hydrates [11,12,20,27]. Pseudopotentials of the norm-conserving Troullier-Martins type were used with a double-zeta polarized basis set and a 10 Å K-grid cut-off. An energy shift of 50 to 100 meV, a mesh cut-off of 800 to 2000 Ry, and a force tolerance of 0.0005 to 0.004 eV/Ang were used. Values of these parameters vary based on the sH hydrate structure being simulated. Tight parameters were necessary for some systems, especially those containing methane as to assure that the optimized structure was a true energy minimum with an IR spectrum that was free from any imaginary frequencies. To investigate sH gas hydrate structures under pressure, hydrostatic pressure was directly applied to the unit cell in the DFT code and the structure was relaxed to minimize its energy under the specified pressure value.

The computations of the IR vibrational frequencies and intensities were completed using the Vibra utility package and the computation details were highlighted by Fernandez-Torre et al. [57]. The finite differences method was used to compute Born charges and force constants using atomic displacement in SIESTA [53]. Each atom in the unit cell was displaced in six directions (±X, ±Y, ±Z) by a fixed value of 0.01 Å in agreement with other studies [20,57]. Macroscopic polarization was used to compute the Born charges using a polarization grid. For the empty sH hydrate, ten points for the line integrals and three points for the surface integrals were used. While for filled systems seven points for the line integrals and three points for the surface integrals were used. Other studies [20,57] have used two points for the line integrals and one point for the surface integrals. In this work, the number of used points was higher to increase the accuracy of the computations. IR vibrational frequencies and intensities were then obtained using the diagonalization of the dynamical matrix. In the Vibra utility package, the IR frequencies and normal modes were evaluated at the gamma **Γ** point as it is the k-point essential for infrared absorption evaluation, which agrees with previous computational IR studies [13,20,57,58].

**Figure 11:**
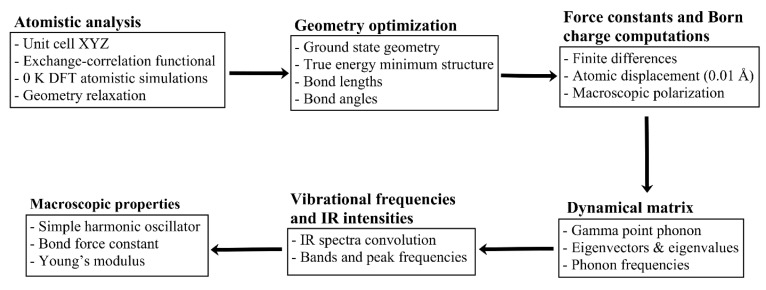
Computational platform formulated and executed in this work. Process to transform microscopic/vibrational analysis inputs into macroscopic properties’ output of the most important and representative sH gas hydrates.

Obtained raw IR spectra are then convoluted using a Gaussian function with a full width at half maximum (FWHM) of 50 cm^−1^ that was suitable for the convolution of most of the bands in the IR spectra of sH gas hydrates. Lorentzian and Gaussian functions were tested, and Gaussian was selected for the convolution of IR spectra in this work. Some vibrational guest bands required the use of a FWHM of 24 cm^−1^ for their convolution. The value of FWHM mainly affects the band’s width with minimal effect on the band’s peak position. In previous studies [20,57], two different values of FWHM for the convolution of IR spectra have been used. The flow chart in Figure 11 presents a summary of the methodology followed in this work.

## 4. Conclusions

Understanding the full set of gas hydrates’ properties is crucial for their utilization and management in different large-scale applications. Structure-H gas hydrate of neohexane and different guest molecules is studied in this work from an IR molecular vibrations perspective. The inclusion of sH hydrates encapsulating carbon dioxide and methane guests is critical to broaden the overall fundamental understanding of this material, which serves its deployment in prospective energy and environmental applications.

The vibrational IR spectra and the findings obtained from its interpretation defines a material’s fingerprints such as structure, composition, and stress load conditions (i.e., pressure). It is one of the useful tools in analyzing gas hydrates’ structures. Using DFT-based IR computations reveal a wide range of useful information about sH gas hydrates, which are related to their vibrational signatures and mechanical characteristics. The link between microscopic vibrations and hydrates’ Young’s modulus emphasizes the fact that hydrogen bonds contribute and even control sH hydrates’ macroscopic properties. Analyzing the vibrational characteristics of sH hydrates under applied hydrostatic pressure provides a better understanding of the pressure dependency of sH hydrate’s stiffness. It also shows the relations between hydrates’ interatomic distances, hydrogen bond strength, and vibrational band frequency shifts as affected by guest type and guest-host interactions. The IR spectra of sH gas hydrates investigated in this work at equilibrium and their vibrational behavior under pressure agree with literature findings. Elastic properties obtained using IR frequencies fall in the same range of those obtained using hydrates’ elastic constants and polycrystalline relations.

The validity of the method used in this work was confirmed by the mechanical data from direct numerical simulations of energy-strain from our previous work [12]. This emphasizes that the transformation of IR spectra into mechanical properties is appropriate for investigating sH gas hydrates. The method has its challenges when dealing with sH hydrate structures containing methane. Such systems might require very strict simulation parameters and several optimization steps to reach a true energy minimum structure with an acceptable IR spectrum. Another challenge could come from the shift in guest molecules’ vibrations (relative to their gas phase). To overcome this challenge, a database of computational-based IR spectra of several hydrates is vital to look at the guest vibrations’ range and factors, which could affect their peak frequencies such as cage shape, cage size, and guest-host interactions.

Discrepancies from temperature and anharmonic effects are encountered by DFT-based IR spectra computations that are based on harmonic approximations [59]. The absence of anharmonicity effects in computations has its impact on the obtained results [57]. In DFT simulations, the roles of exchange-correlation functional and used pseudopotentials are critical in determining the investigated structure properties. Studying the effect of different DFT simulation parameters is essential to identify the best set of parameters that can be used to describe the structure’s vibrational and mechanical characteristics.

Using ab initio DFT to obtain the IR spectra of the hydrogen bonded structure-H (sH) gas hydrate adds to the knowledge and available databases of hydrate spectra and properties specially where experimental IR and mechanical analysis are inaccessible. The findings of this work contribute to the science of gas hydrates with useful data for the prediction of the sH hydrate structure, its composition, and mechanical conditions, which can distinguish it from liquid water, ice, and other hydrates and materials.

## Figures and Tables

**Table 1:** Structural characteristics of structure-H (sH) hydrates, a-lattice and c-lattice constants, unit cell volume (V), oxygen-oxygen (O···O) distance, hydrogen bond (O···H) length, and the covalent bond (O-H) length at 0 K and 0 GPa.

sH Hydrate	a, Å	c, Å	V, Å^3^	O···O, Å	O···H, Å	O-H, Å
Empty	11.93	9.76	1206.15	2.702	1.698	1.009
CH_4_-NH	12.33	10.12	1330.95	2.792	1.797	1.000
Xe-NH	12.48	10.10	1349.53	2.806	1.810	1.000
CO_2_-NH	12.86	9.88	1350.21	2.808	1.813	0.999
CO_2_-CH_4_-NH	12.37	10.05	1334.05	2.795	1.799	1.000

**Table 2:** Convoluted IR spectra peak frequencies (cm^−1^) of host (H_2_O) vibrations in sH gas hydrates at equilibrium (0 K, 0 GPa).

sH Hydrate	Hbond Stretch	H_2_O Libration		H_2_O Bending	OH Sym. Stretch	OH Asym. Stretch
Empty	223	703	1009	1640	2954	3204
CH_4_-NH	186	657	904	1636	3067	3269
Xe-NH	186	645	892	1626	3119	3314
CO_2_-NH	182	615	886	1629	3102	3283
CO_2_-CH_4_-NH	183	622	898	1635	3067	3272

**Table 3:** Convoluted IR spectra peak frequencies (cm^−1^) of guest vibrations in sH gas hydrates at equilibrium (0 K, 0 GPa).

sH Hydrate	C-C-C Bend	CO_2_ Bend	CH_2_/CH_3_ Rock	CH_4_ Bend	CH_3_ Sym. Bend	CH_3_ Asym. Bend	CH_4_ Rock	CO_2_ Asym. Stretch
CH_4_-NH	408/474	-	1196	1252	1344	1431	1478	-
Xe-NH	406/483	-	1209	-	1347	1435	-	-
CO_2_-NH	404/480	*	1203	-	1344	1430	-	2284
CO_2_-CH_4_-NH	408/474	*	1196	1251	1344	1430	1478	2284

* Only observable under compression.

**Table 4:** Hydrogen bond force constant (**k**) and corresponding sH hydrate Young’s modulus (**E**) from hydrogen bond stretching frequency.

sH Hydrate	k, N.m^−1^	E (IR Analysis), GPa	E (Elastic Constants), GPa [12]
Empty	2.778	16.36	14.317
CH_4_-NH	1.933	10.76	16.569
Xe-NH	1.933	10.68	17.002
CO_2_-NH	1.850	10.20	-
CO_2_-CH_4_-NH	1.871	10.40	-

**Table 5:** Pressure dependence of guest vibrational frequencies in sH gas hydrates.

Guest Vibrational Frequencies, cm^−1^								
**Pressure, GPa**	C-C-C Bend	**CO_2_ Bend**	**CH_2_/CH_3_ Rock**	**CH_4_ Bend**	**CH_3_ Sym. Bend**	**CH_3_ Asym. Bend**	**CH_4_ Rock**	**CO_2_ Asym. Stretch**
**Xe-NH sH Hydrate**								
−1	404/479	-	1204	-	1349	1437	-	-
−0.5	405/480	-	1207	-	1348	1434	-	-
0	406/483	-	1209	-	1347	1435	-	-
1	408/488	-	1214	-	1350	1439	-	-
2	491	-	-	-	1353	1442	-	-
2.9	494	-	-	-	1353	1443	-	-
4	-	-	-	-	1354	1447	-	-
**CO_2_-NH sH Hydrate**								
−0.955	403/475	-	1197	-	1343	1428	-	2270
−0.5	403/477	-	1201	-	1344	1429	-	2278
0	404/480	-	1203	-	1344	1430	-	2284
1	404/482	612	1206	-	1346	1432	-	2292
2	483	609	-	-	1346	1433	-	2299
3	486	607	-	-	1347	1435	-	2304
**CO_2_-CH_4_-NH sH Hydrate**								
−0.5	405/472	-	1194	1250	1343	1429	1477	2277
0	408/474	-	1196	1251	1344	1430	1478	2284
2	478	611	1199	1255	1345	1433	1483	2301
3	479	610	-	1253	1346	1434	1484	2308

**Table 6:** Pressure dependence of hydrogen bond force constant (**k**) and Young’s modulus (**E**) of sH gas hydrate.

Empty sH	**Pressure, GPa**	**k, N.m^−1^**	**E, GPa**
	−1.405	0.778	3.92
	−1	1.691	9.30
	−0.5	2.190	12.53
	0	2.778	16.36
	1	3.864	23.65
	2	4.194	26.48
	3	5.685	36.81
Xe-NH sH	−1	1.003	5.16
	−0.5	1.308	7.02
	0	1.933	10.68
	1	2.394	13.79
	2	3.164	18.81
	2.9	4.012	24.38
	4	4.164	25.90
CO_2_-NH sH	−0.955	0.652	3.27
	−0.5	1.342	7.15
	0	1.850	10.20
	1	2.704	15.59
	2	3.381	20.09
	3	3.747	22.81
CO_2_-CH_4_-NH sH	−0.5	1.325	7.11
	0	1.871	10.40
	2	3.548	21.26
	3	3.983	24.47

## Supplementary Materials

The following are available online, schematics of sH gas hydrate water molecules’ main vibrational modes as well as some guest vibrations are available with average frequency values.
