# Supplementary material for: From Infrared Spectra to Macroscopic Mechanical Properties of sH Gas Hydrates through Atomistic Calculations
Source: Molecules. 2020 Nov 27;25(23):5568. doi: 10.3390/molecules25235568 (PMC7729739; doi:10.3390/molecules25235568)
Supplement: Supplementary file 1 [file molecules-25-05568-s001.pdf]

From Infrared Spectra to Macroscopic Mechanical Properties of sH Gas Hydrates through Atomistic Calculations

Supporting Information

Authors: Shaden M. Daghash[a], Phillip Servio[a], Alejandro D. Rey[a]*

[a] *Department of Chemical Engineering, McGill University, Montréal, Quebec H3A 0C5, Canada*

*Corresponding author email: alejandro.rey@mcgill.ca

Content:

This file contains schematics of the main vibrational modes of water molecules and some guest vibrations in sH gas hydrates with average IR frequency values at 0 K and 0 GPa (unless otherwise stated). Molecules visualizations were made using VESTA* software.

Color code:

- Oxygen atom: red
- Hydrogen atom: white
- Carbon atom: brown
- Vibrations happening at the same time have the same color (black or blue).

***** K. Momma and F. Izumi, "VESTA 3 for three-dimensional visualization of crystal, volumetric and morphology data," J. Appl. Crystallogr., 44, 1272-1276 (2011).

Main vibrational modes of water molecules in sH gas hydrates

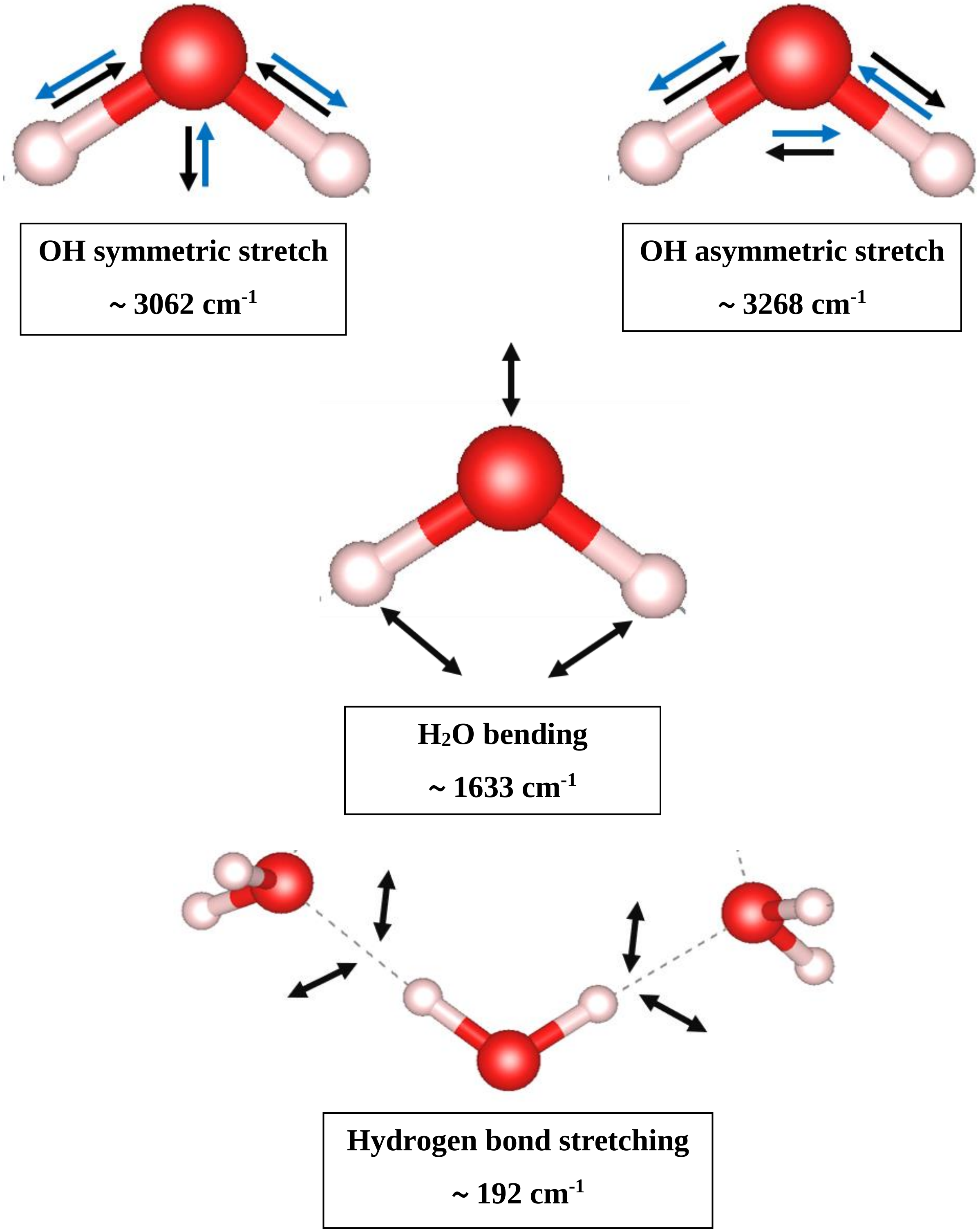

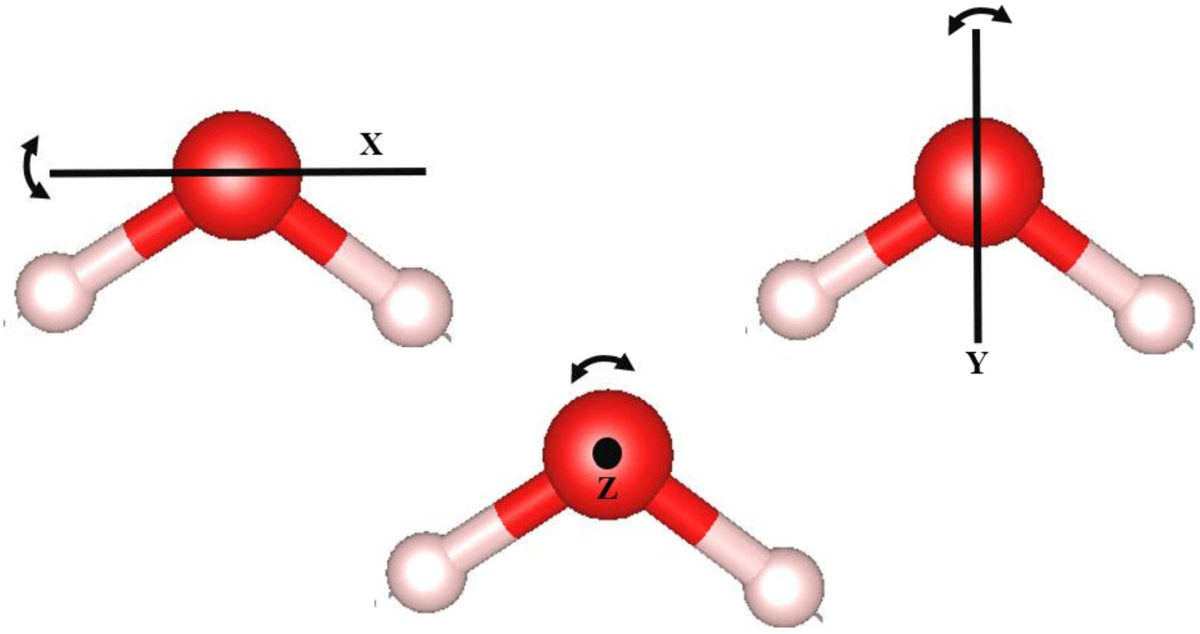

H2O libration modes

~ 648 cm^{-1}, 918 cm^{-1}

Selected vibrational modes of guest molecules encapsulated inside sH gas hydrates

Carbon Dioxide

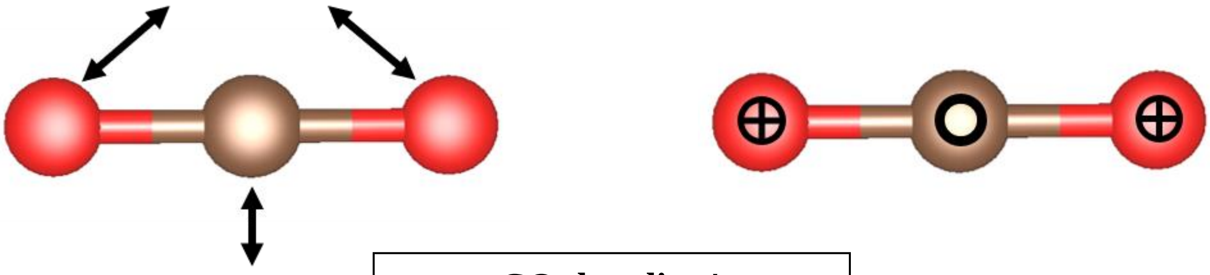

CO_2 bending*

~ 610 cm^{-1}

*** Observed at pressures ≥1 GPa in sH gas hydrate**

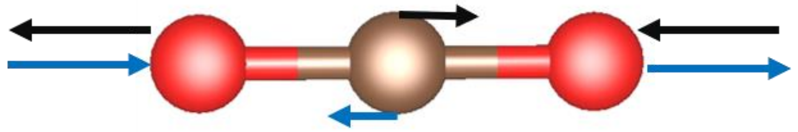

C=O asymmetric stretch

~ 2284 cm^{-1}

Methane

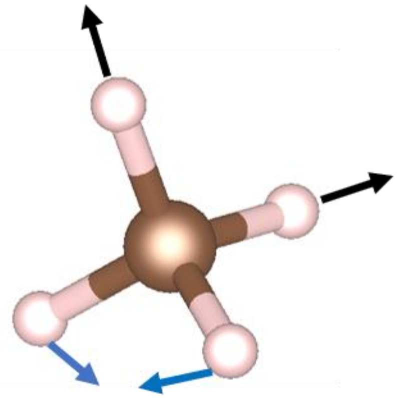

CH_4 asymmetric bending

~ 1252 cm^{-1}

Neohexane

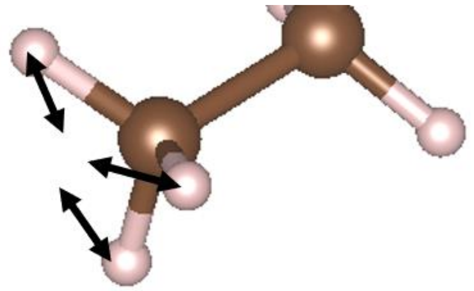

CH_3 symmetric bending

~ 1345 cm^{-1}

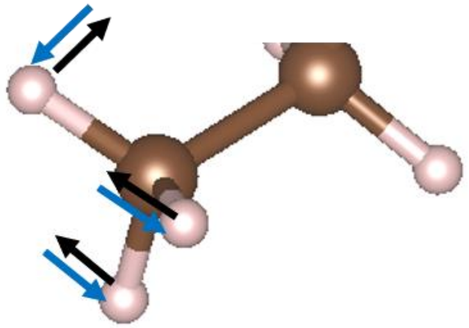

CH_3 asymmetric bending

~ 1432 cm^{-1}
